# **Creating an emission model based on portable emission measurement system for the purpose of a roundabout**

**DOI:** 10.1007/s11356-019-05264-1

**Published:** 2019-05-25

**Authors:** Artur Jaworski, Maksymilian Mądziel, Kazimierz Lejda

**Affiliations:** Faculty of Mechanical Engineering and Aeronautics, Department of Combustion Engines and Transport, Rzeszow University of Technology, 8 Powstancow Warszawy Ave, 35-959 Rzeszow, Poland

**Keywords:** Air pollution, Roundabout emission modeling, Vehicle emission, Exhaust measurements, Portable emissions measurement systems (PEMS)

## Abstract

Road transport is the main source of pollution to the environment in urban areas; therefore, there is a need to accurately estimate the amount of exhaust gases emitted by motor vehicles. The development of systems for measuring emissions of exhaust gases caused the exit from stationary chassis dynamometers to real road test. This paper presents an analysis of emission data from the PEMS system for real driving cycles of various types of vehicles, complying with EURO2-EURO6 standards, fueled with petrol, LPG, and diesel in urban, rural, and motorway areas as well as detailing roundabouts. The results show that in the range of roundabouts, there is an increased emission of harmful exhaust components, such as CO_2_, THC, CO, and NO*x*. Due to the specific traffic conditions that prevail at the roundabout (acceleration, braking, acceleration to a certain speed), the methodology for creating an exhaust emission model for this type of objects has been proposed. Statistical analysis of the received boosted regression tree models based on the coefficient of regression, root mean square error, and mean absolute error and based on the visual assessment of the results show that the obtained models are well represented by real data. The obtained results of emission calculations on roundabouts may be used to identify areas of increased emission of harmful exhaust components, as well as an introduction to prepare new roundabout design guidelines concerning emission data.

## **Introduction**

Nowadays, climate change is believed to be the second most important problem faced by the global community (European Commission 2011). The main greenhouse gases emitted by human activities are carbon dioxide, methane, nitric oxide, hydrofluorocarbons, perfluorocarbons, and sulfur hexafluoride (Venkataraman et al. 2012; Wei et al. 2008; Lindley, McCulloch 2005).

Transport is the main sector of the economy that causes environmental pollution and climate change. Emissions from transport, mainly road transport, make a significant contribution to the number of greenhouse gases in the atmosphere (OECD 2002). The automotive industry is the second largest sector producing CO_2_ with a total share of 22% in general (IEA 2012).

The World Health Organization estimates that around 2.4 million people lose their lives worldwide due to environmental pollution (WHO 2007). In addition to greenhouse gas emissions from vehicles, emissions of particulate matter, carbon monoxide, and hydrocarbons are directly dangerous to the natural environment and man. Of all components of the exhaust gases, the largest, direct, negative impact on human health has particulate matter, mainly those with a diameter of 10 μm and less. The share of its emission by motor vehicles, especially in winter, contributes to the formation of London-type smog. Such phenomena increase the number of people suffering from lung cancer, chronic obstructive pulmonary disease, and cardiovascular diseases (WHO, 2018).

Polish air in terms of pollution is one of the worst in Europe (EEA, 2016; Wdowiak et al., 2018), and therefore it should strive to minimize the adverse impact on the environment by motor vehicles, through continuous monitoring of the current state and implementation of, e.g., new infrastructural solutions that are more environmentally friendly.

Due to the fact that the permissible exhaust emission limits to air quality standards are being exceeded more often, there is a need to develop new models for calculating emissions from road transport, to properly estimate the impact of vehicle traffic on the overall emission of harmful exhaust components. In order to adequately reflect actual conditions, the effects of emissions on a local scale should be taken into account, using modern technologies for measuring emissions. In order to estimate the impact of, for example, intelligent traffic control systems, dynamic speed limits, or different types of intersections (roundabouts) on emissions, calculations should be sufficiently sensitive to the characteristics of vehicle traffic (Noland, Quddus, 2006; Smit et al., 2010).

US, German, or Polish conditions and guidance to design new roundabout minimalize the emission factor; these documents describe the general environmental conditions, but don’t give approximate results of emission from cars crossing this kind of intersection (FHA 2000; Tracz et al., 2001; FGSV 2006; FHA 2010). This publication will be an introduction and proposition to create new roundabout design guidance policy in aspect of emission.

## 2. Background

### **Vehicle emission models**

Classification of road traffic models and emissions of pollutants is divided, due to the accuracy scale: macroscopic (regional), mesoscopic (local), and microscopic (intersections, sections of streets).

Existing emission models are distinguished into two categories:

- Models that use road traffic parameters such as acceleration, braking, continuous driving, and idling
- Models that are based on the average speed parameter

The more detailed classification lists the models:

- Type 1—models that previously have loaded speed profiles
- Type 2—models that generate speed profiles as part of the modeling process
- Type 3—models that require speed profiles to be loaded

Macroscopic models are based mainly on the average speed parameter on the analyzed sections of the road network (Zhu 2014; Akcelik and Besley 2003). They are used to estimating fuel consumption and environmental impacts by road transport in order to determine the impact of total energy consumption by projects and road infrastructure development strategies and assess the impact of greenhouse gas emissions on the researched area.

One of the exemplary programs that contain models for calculating exhaust emissions on a macroscale is the Operat FB. It has a vehicle module that makes it possible to calculate emissions from traffic. Pollution emission results are calculated in accordance with the EMEP/Corinair B710 and B760 methodology. It consists in calculating the hot emission from vehicle engine exhaust, cold emission generated at the beginning of the engine operation, and emission of evaporation. It is also possible to calculate the emission coming from abrasion of brake pads and tires (Manual OperatFB, 2015). The Corinair model distinguishes over 200 categories of vehicles, separated into 6 groups: passenger cars, vans, lorries, buses, mopeds, and motorcycles (Jaworski et al., 2018a).

The model that allows calculating emissions for US data is MOBILE, developed by the US EPA. It uses data collected by EPA, CARB, and automotive manufacturers, as well as traffic inspection tests collected in various states. Emission for specific vehicle classes is a product of traffic volume and emission factors. The methodology used to calculate the emission takes into account a number of factors calibrating the model, such as the surrounding environment, vehicles used, and their operational status (EPA, 2006).

Microscopic models of emission calculations require many data based on continuous measurement of basic vehicle parameters, such as speed, acceleration, terrain gradient, and position coordinates. They calculate emissions continuously in a specific unit of time, usually 1 s.

The general classifications of emission models of exhaust emissions in the microscale due to the data used covers (Leung, Williams, 2000; Barth, Scora, 2006; Rakha et al., 2011; Ahn et al., 2002; Int Panis et al., 2006; Qi et al., 2004; Smit et al., 2008; Smit, McBroom 2009; Ligering and Lange, 2012):

- Based on the speed profile: VT, Versit +
- Based on vehicle power: CMEM, VT-CPFM, CSIRO
- Hybrids of the above: models developed by Smit, McBroom, and Qi

The VERSIT + emission model, which is used in the Enviver program, is a multifactorial regression model in which the driving cycle of a given vehicle is variable. This requires earlier obtaining speed profiles in the Vissim program, and later usage of them to estimate the emission factors (g/km) for different vehicle classes (Smit et al. 2006; Smit and McBroom, 2009). In contrast to the emission factors obtained from the New European Driving Cycle (NEDC), the speed profiles used in this model are representative of the real road conditions (Rexeis and Hausberger 2009; Jaworski et al., 2017b). Emission factors (EFj, k, l) are obtained from multiple linear regression to find empirical relationships between the degree of emission, velocity profile, and dynamic variables (Smit et al. 2008).

An example of a model based on vehicle power is CMEM, which was developed in 2006. Emission processes are divided into different categories that correspond to physical phenomena related to vehicle operation (Barth et al., 1996). When developing these models, emissions were measured both at the engine and at the exhaust outlet of the tested vehicle. In total, over 300 vehicles were tested in laboratory conditions in three driving cycles. However, the development of this type of model requires a lot of data. To calculate real-time emissions, many physical variables must be collected, and a vehicle speed profile is required as input.

### **Modeling of emission for roundabout purposes**

In the literature presented so far, few publications on exhaust emissions from vehicles can be found on various solutions of roundabouts (Abhisek et al. 2014; Yperman, Immers, 2003; Engelsman, Uken, 2007). Studies related to the comparison of other parameters such as capacity, driving safety, and delays that occur at roundabouts are more common (Fortuijn, 2009; Giuffrè, et al., 2009; Mauro, Branco, 2010; Giuffrè, et al., 2012; Silva et al. 2014; Vasconcelos et al., 2014a; Mauro, Cattani, 2010).

Most of the work devoted to exhaust emissions at roundabouts relates to the use of already-existing models for calculating exhaust emissions. In this respect, the use of computational models is significantly limited, as macroscale models are not suitable for testing objects on a microscale such as roundabouts.

One of the most widespread emission models for calculations on a microscale, including roundabouts, is the VSP (vehicle-specific power) model. It uses data from PEMS systems installed in tested cars under US conditions. On this basis, this model requires the input of speed, acceleration, and gradient to calculate the emission of harmful exhaust components generated by vehicles (Frey et al., 2008, Kutz, 2008). Examples of using this method are articles (Coelho et al., 2006; Salamati et al., 2013; Anya et al., 2013; Mudgal et al., 2014; Vasconcelos et al., 2014b), which present the assessment of exhaust emissions from vehicles for various roundtrip schedules. The authors of the study (Coelho et al., 2006) present three characteristics of the speed profile for a vehicle traveling to a single-band roundabout ((1) without stopping, (2) with one stop, (3) with several stops). This study describes the occurrence of these profiles depending on the intake and crossing of traffic flows. Based on their findings, the authors developed regression models to describe the occurrence of these speed profiles for a single-band roundabout. The extension of this research is a study (Salamati et al., 2013), which also includes conventional two-lane roundabouts. However, the article (Anya et al., 2013) presents the environmental differences that resulted from the conversion of the intersection with traffic lights to a two-lane roundabout.

The authors of the paper (Jaworski et al., 2017a) compared exhaust emissions during driving on a two-lane and turbine roundabout, based on microsimulation models and real traffic data. The Enviver model was used to assess emissions of exhaust gases, which allows the calculation of emissions on a microscale. The results show that the peak hour emission for the turbine roundabout is about 1/3 lower compared to the two-lane roundabout. Unfortunately, the results were limited only to the comparison of two toxic components of exhaust fumes: NO*x* and PM10.

Attempts to assess emissions during driving around the roundabout using the PEMS system were made by the authors (Liu et al., 2017; Gastaldi et al., 2017; Meneguzzer et al., 2017). In study (Liu et al., 2017), the authors undertook the assessment of exhaust emissions at the inlets to single-band roundabouts with the use of PEMS and OBDII systems. The main limitation of this work was driving using only one vehicle and measuring only CO_2_. Papers (Gastaldi et al., 2017; Meneguzzer et al., 2017) refer to the comparison of the originally functioning intersection with traffic lights to the modernized intersection of the roundabout. The authors made 396 trips through both intersections using two different drivers and for a different time of day. Studies have shown reduced CO_2_ emissions for a roundabout compared to an intersection with traffic lights. On the other hand, the NO*x* emission was increased to the disadvantage of the roundabout.

The above works concerned the emission testing at the roundabout based on previously created general emission models. However, due to the fact that traffic on roundabouts has a specific characteristic, there is a need to create emission models for exhaust gases for roundabouts based on road and real data. An additional premise for the creation of new emission models for Poland and Eastern and Central European countries is a larger number of models of older types of vehicles, which additionally generates errors in estimating emissions using the model and western ones due to the different technical condition of vehicles.

## **Research methodology**

### **Experimental equipment**

To do the research part, the PEMS system at the Department of Combustion and Transport Engines at the Rzeszow University of Technology was used. It is equipped with the following sensors and methods for measuring harmful components in the flue gas: a flame ionization detector (FID) for HC measurement, a non-dispersive infrared spectrometer (NDIR) for CO and CO_2_ measurement, chemiluminescence (CLD) for measuring NO and NO_2_. Accordingly, to the EU Commission Regulation 2017/1151, acceptable deviations of PEMS measurements compiled with results from a stationary analyzer (on a chassis dynamometer) should not be greater than 15% for HC, CO, NO*x*, and 10% for CO_2_ (Commission Regulation UE, 2017; Jaworski et al., 2018c). PEMS can be installed in the boot of the tested vehicle, while the measuring sensors with a flow meter are connected to the outlet pipe (Varella et al., 2019). The exhaust gas pipe must be heated to 190 °C to avoid condensation of hydrocarbons. In addition, ambient temperature and humidity sensors, as well as a GPS transmitter, are connected to the system. In order to get a full picture of the impact of the engine operation on the generated emission, the OBDII interface (Weiss et al., 2011; Ligterink, 2016; Giechaskiel et al. 2016) can be connected to the ECU of the vehicle. Selected technical parameters of the PEMS system used for testing are presented in Table 1.

**Table 1:** Selected technical parameters of the PEMS Horiba OBS-2200 system

Parameter	Measurement method	Accuracy
The concentration of exhaust components:		
CO	NDIR—non-dispersive (infrared), range 0–10%	± 2.5%
CO_2_	NDIR—non-dispersive (infrared), range 0–10%	± 2.5%
THC	FID—flame ionization, range 0–10000 ppm	± 2.5%
NO_*x*_	CLD—chemiluminescence, range from 0–100 to 0–3000 ppm	± 2.5%
Sampling frequency	1 Hz	
The heating time of the analyzers	Up to 1 h	
Gas flow	Mass flow rate	In the range of ± 1.5% of full scale or within ± 2.5% of readings

**Fig. 1:**
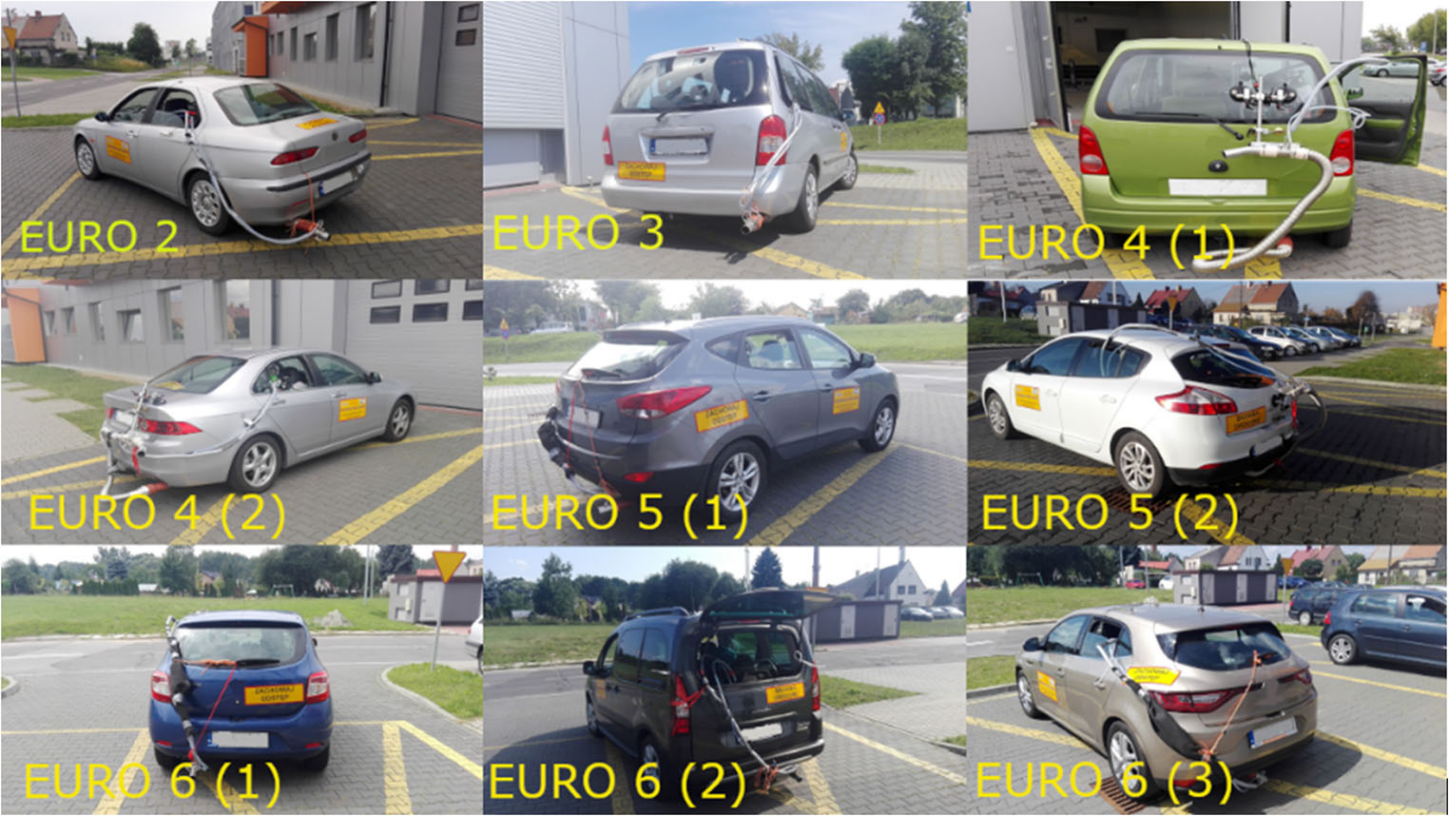
Vehicles used for road cycles

Most of the cars driving through researched roundabouts were passenger cars so that in this paper only this class of vehicle was used. To conduct the work, passenger cars presented in Table 2 were used, while their pictures are shown in Fig. 1.

**Table 2:** Selected technical parameters of the tested cars

Parameter	The emission standard								
	Euro 2	Euro 3	Euro 4 (1)	Euro4 (2)	Euro 5 (1)	Euro 5 (2)	Euro 6 (1)	Euro 6 (2)	Euro 6 (3)
Production year	1998	2001	2003	2004	2011	2012	2014	2018	2017
Engine capacity (cm^3^)	1598	1991	1199	1998	1591	1397	1149	1560	1197
Engine type	Spark ignition	Spark ignition	Spark ignition	Spark ignition	Spark ignition	Spark ignition	Spark ignition	Diesel	Spark ignition
Fuel type	Gasoline	Gasoline/LPG	Gasoline	Gasoline	Gasoline	Gasoline.	Gasoline/LPG	Diesel oil	Gasoline
Max. power (kW)/with speed (rpm)	88/6300	90/5800	55/5600	115/6000	99/6300	96/5500	55/5500	88/3500	74/4500
Max. torque (Nm)/with speed (rpm)	144/4500	175/4500	110/4000	190/4500	164/4850	190/2250	105/4250	300/1750	175/1500
Gearbox/number of gears	Manual/5	Manual/5	Manual/5	Manual/5	Manual/6	Manual/6	Manual/5	Manual/6	Manual/6
Power system	MPI	MPI	MPI	MPI	GDI	MPI	MPI	CR	MPI
After-treatment system	TWC	TWC	TWC	TWC	TWC	TWC	TWC	DPF+ SCR+ DOC	TWC
Weight (kg)	1230	1600	1040	1430	1305	1280	980	1429	1205

All vehicles tested before the tests were checked using stationary exhaust gas analyzers in accordance with the polish test procedure of periodic diagnostic tests. The results of concentrations and amounts of harmful exhaust components of the tested vehicles were within acceptable limits. The car complying with the EURO 2 standard before the start of the test was 178,000 km; EURO 3 260,000 km; EURO 4 (1) 21,000 km; EURO 4 (2) 85,000 km; EURO 5 (1) 126,000 km; EURO 5 (2) 145,000 km; EURO 6 (1) 42,000 km; EURO 6 (2) 20,000 km; and EURO 6 (3) 36,000 km.

Such choice of vehicles is justified by the fact that according to statistical data, the number of passenger cars meeting EURO standards for road conditions in Poland in 2020 for EURO 2 will amount to approx. 12%, EURO 3 25%, EURO 4 28%, EURO 5 20%, and EURO 6 10%. The following years, and consequently the introduction of further restrictive regulations regarding the entry of city cars with internal combustion engines, will increase the number of new vehicle structures and in 2030 the percentage of vehicles complying with the EURO 6 standard should amount to approx. 30% (Website of the Polish Local Data Bank, 2018).

The work is focused on passenger cars because the main part refers to infrastructure facilities inside cities, where the number of passenger vehicles is dominant.

### **Experimental campaign**

**Fig. 2:**
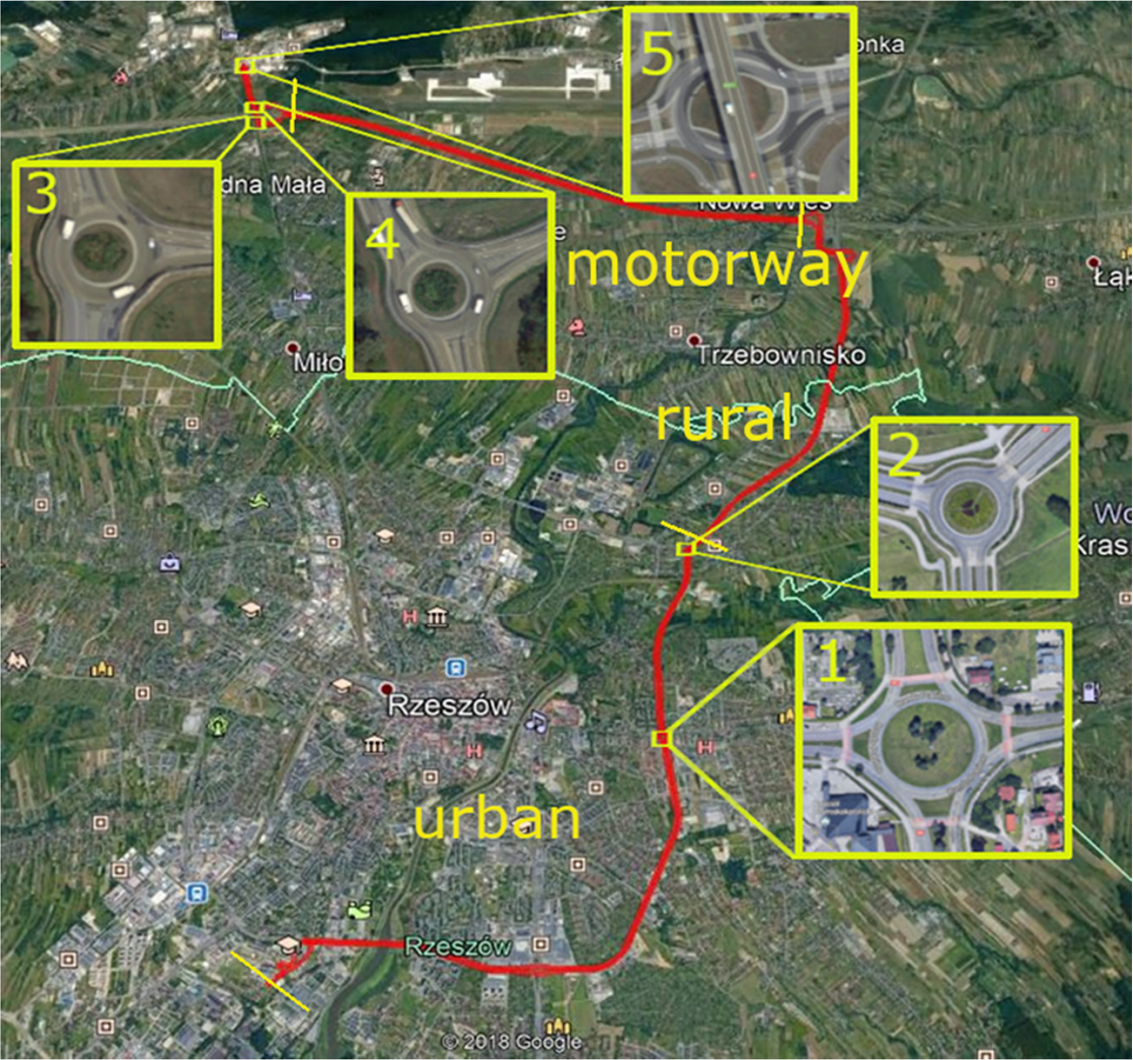
The chosen route (red line) with marked roundabouts; yellow lines indicate the beginning/end of particular part of the route

In order to collect the emission data of harmful exhaust components in the first stage of the works, the route on which the emission values were recorded was selected. Data from the real trip were used to create emission models for selected exhaust components. A large amount of input data was needed to create them, which is why the route was 40 km long and included crossing through 5 roundabouts. The created models are based not only on the passage sections through the roundabout, but also include sections of arrivals and departures from it, which is why the speed range taken into account when creating models included values from 0 to 80 km/h. The route including urban, rural, and motorway sections was chosen to obtain a full cross-section of vehicle emission data under different road conditions. The vehicles for each fuel type were tested on the researched road parts 2 times. Trials were made in 2017/2018 in comparable ambient conditions. Each attempt lasted about an hour. The PEMS OBS-2200 system automatically registered emission data and vehicle operational data. In addition, the OBD II system was connected to vehicles in order to later verify the speed data to the PEMS system GPS data. The data was recorded at a frequency of 1 Hz so that each attempt generated about 30,000 records. All data record contained information about speed, acceleration of the vehicle, amount of fuel used, emission level of the exhaust components tested, humidity, air temperature, and GPS coordinates. The route chosen for the study is shown in Fig. 2.

Selected parameters of researched roundabouts are presented in Table 3. As a result of the differences resulting from the description of the geometry of the two-lane and turbine roundabout, the outer diameter of the turbine roundabout was given approximately, in order to match it with the geometry of the remaining researched roundabouts.

**Table 3:** Parameters of the researched roundabouts.

No.	Type of roundabout	The outer diameter of the roundabout (m)	The diameter of the central island (m)	Number of inlets/outlets
1	Two-lane	87	69	4
2	Two-lane	66	44	3
3	Two-lane	45	25	3
4	Two-lane	45	25	3
5	Turbo	≅ 62	–	4

### **Modeling exhaust emissions with the use of statistical tools**

**Fig. 3:**
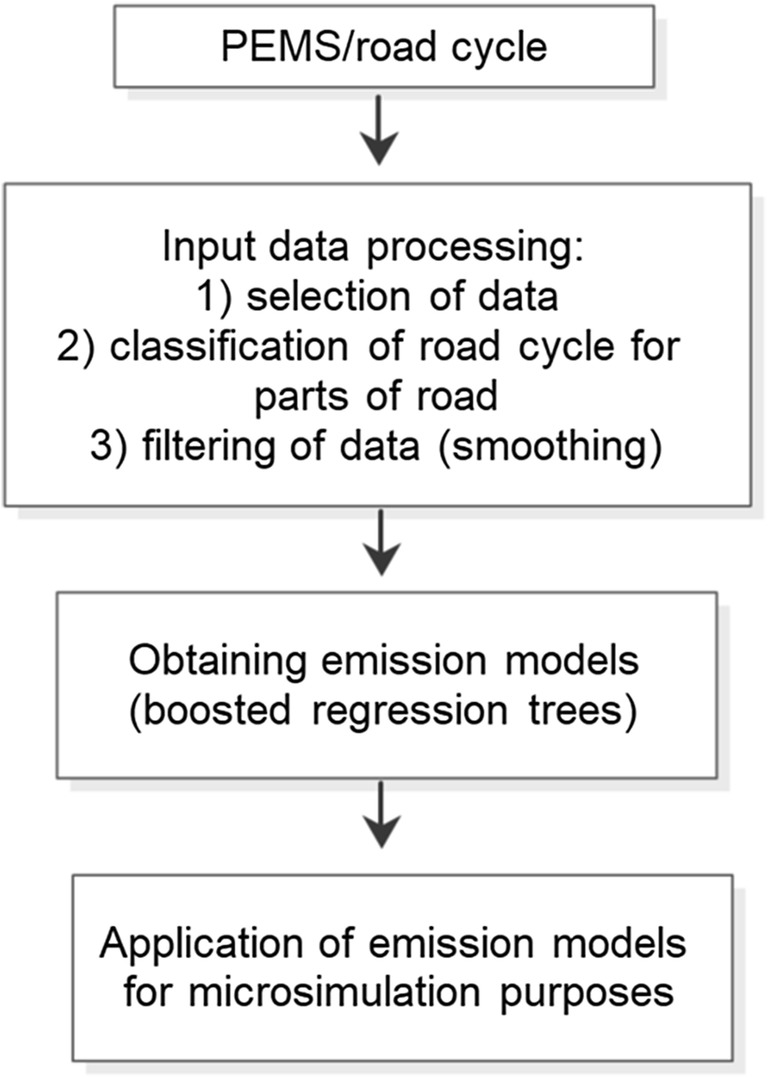
The general structure of the issue model construction for roundabouts.

The data from the trip was limited to data from the area of roundabouts (300 m) and roundabouts; therefore, the speeds to be analyzed range from 0 to 80 km/h, while the acceleration/deceleration is on average from − 3 to 2.8 m/s^2^. The general structure of the construction of the exhaust emission model is shown in Fig. 3.

In order to ensure the validity of the obtained data, some procedures were carried out on data obtained from the PEMS system. Independent variables from the OBD system were synchronized, such as speed and acceleration with emission data of exhaust emissions. There may be some delay in measuring emissions because it takes time to transport exhaust gases from the engine to the exhaust gas analyzers. In addition, the data smoothing technique included in the Matlab program has been applied to reduce unnecessary noise.

The methodology of creating computational emission models of exhaust emissions in the microscale is classified into four basic groups (Barth et al., 1996; Rakha et al., 2004; Guo et al., 2012; Barth et al., 2000):

- Models based on emission maps include tables of collected emission data and fuel consumption, taking into account vehicle speed and acceleration data or engine data such as speed and torque; this emission model may be too sensitive to the vehicle driving cycle data.
- Regression models calculate the emission of individual exhaust components based on mathematical functions, for which the input data are current speeds and vehicle accelerations, sometimes also a gradient of terrain and other auxiliary quantities.
- Models based on engine load is based on a function that contains a series of variables that determine the physical aspects of emissions generated in a vehicle engine, but they require a lot of input data to calculate emissions, which limits their applicability.
- Models based on neural networks require a lot of input data for network learning; these models also need a lot of time to calculate the emission value.

In connection with the above, the paper attempts to create emission models for harmful exhaust components using the regression method.

An important step in creating a regression model is to define a subset of predictors that most affect dependent variables, i.e., harmful exhaust components in the case studied. In order to find a combination of variables that best fit the data, the selection of variables to the model using the method of the best subsets (all-possible subsets) based on the Mallow Cp criterion (Wu and Hamada, 2000) was applied. This algorithm leads to the creation of many models with different regression functions with the maximization of prediction and the minimization of the number of variables.

## **Results and discussion**

### **Results of road tests**

**Fig. 4:**
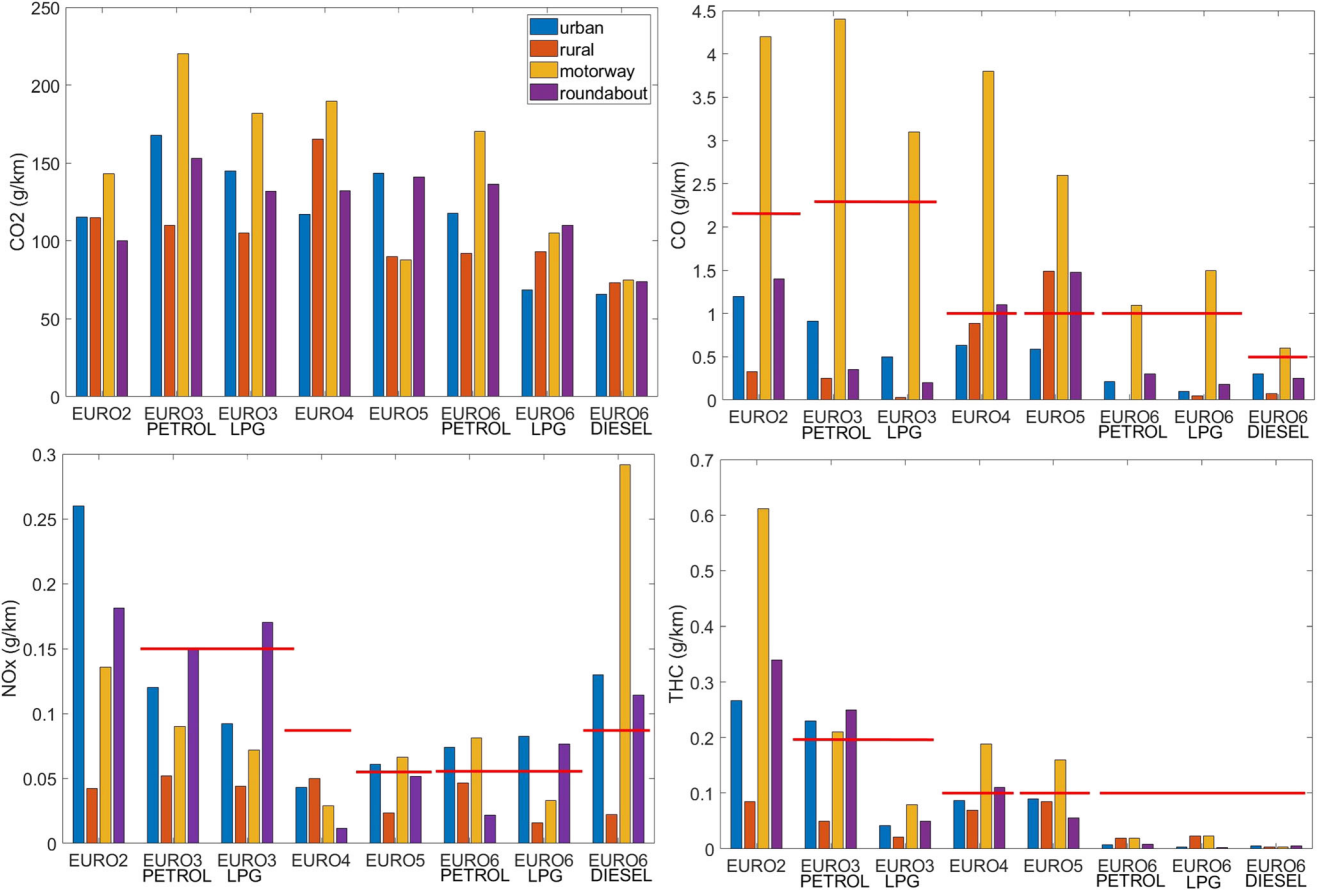
Results of emission of selected exhaust components for excluded parts of the test route (red line shows the limit of EURO standard)

The tests were carried out in 2017/2018 using previously described cars driven by 5 different drivers, under comparable temperature conditions. The test route, due to the specificity of the area and the driving characteristics, was separated into 4 parts: urban, rural, motorway, and roundabouts. Results of CO_2_, THC, NO*x*, and CO emission from crossings for particular parts of the test route are shown in Fig. 4.

Based on Fig. 4, it can be noticed that:

- for most tested vehicles, the highest CO_2_ emission was for the motorway part, which is related to the largest fuel consumption by the engine;
- the smallest emission of CO_2_ is characterized by vehicles complying with the EURO 6 standard, especially those with a diesel engine, which is mainly caused by the lowest fuel consumption;
- CO_2_ emissions occurring at roundabouts are comparable to the average emissions obtained in the urban part; for most EURO standards, the differences from the urban part to roundabouts are about 10–15%; the largest difference is for the EURO6 standard (LPG) and is about 37%;
- exceeding the limit values of THC for EURO standards for the tested vehicles occurred for the EURO3 vehicle (petrol) in the urban, motorway, and roundabouts part, for the EURO4 vehicle in the motorway section and roundabouts, and for the EURO5 vehicle in the motorway section;
- the EURO6 diesel vehicle indicates a significant excess of the NO*x* emission standard, which results from the specificity of the combustion process for diesel engines; only the rural emissions are within the norm; and
- for all vehicles with the exception of EURO4 and EURO5, for the urban and rural part, and roundabouts, there are no exceeded limit values of CO, while for EURO4 and EURO5, for the parts of roundabouts, there are exceedances, which amount to 10 and 48% respectively.

Analyzing in detail the emission data obtained by crossing the roundabout, it can be noticed an increased emission of harmful exhaust components, at the moment of acceleration at entries, and the driving around the island, as well as at the exit points, wherein particular the highest CO_2_ emission occurs. Decreasing the speed or stopping the vehicle and waiting at the entrance to the roundabout is conducive to increased CO, NO*x*, and THC emission at the moment of acceleration.

**Fig. 5:**
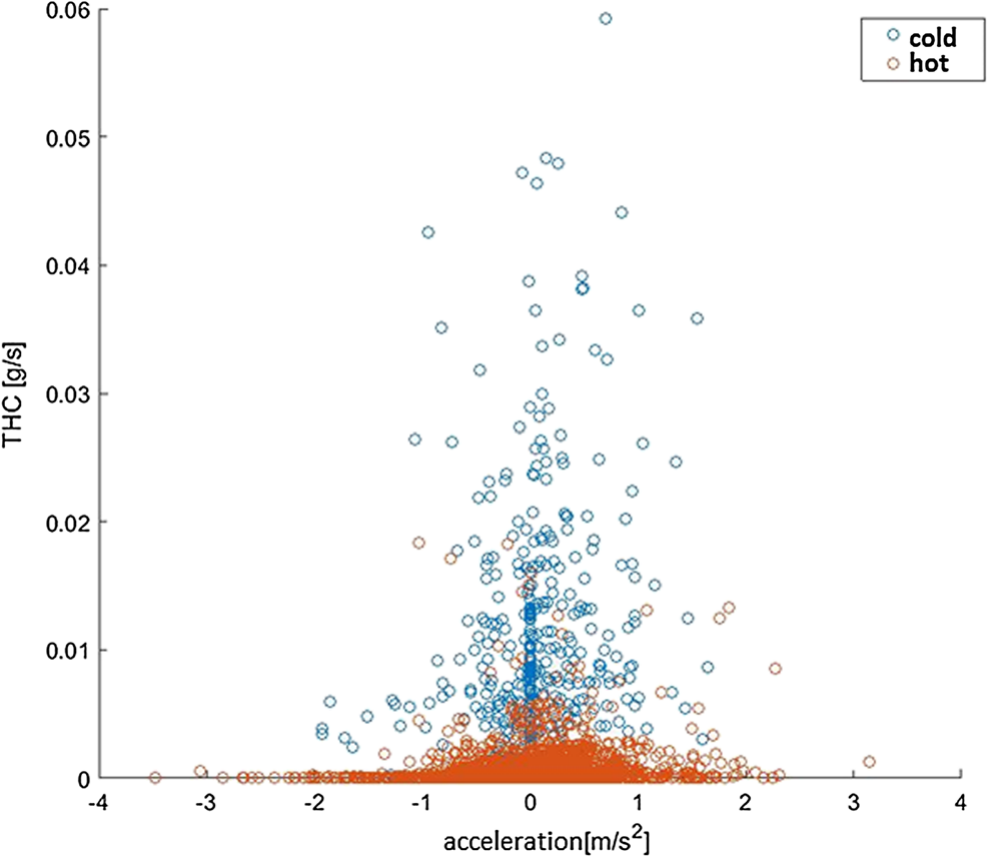
Instantaneous emission THC for the warm and cold thermal conditions of the vehicle engine meeting the EURO2 standard

An additional aspect of modeling exhaust emissions is the problem of cold engine starting. When creating emission models for inner-city areas, a certain percentage of vehicles must be assumed, which is characterized by increased exhaust emissions for cold engine starting. For this purpose, additional emission models for cold engine starting for individual emission classes will be created. Differences in THC emissions that result from driving during a cold start and a warm engine are shown in Fig. 5.

Low temperatures of catalytic compounds, at cold engine start, reduce the efficiency of catalytic reactors (Jaworski et al., 2018b). Increased emission values due to cold engine start occur mainly in urban areas. Emissions of carbon monoxide can be about 50% higher in the event of a cold engine start compared to a hot one. The same applies to hydrocarbons, for which this increase is about 30%. Also, emissions of nitrogen oxides can increase by approx. 100% for SI engines.

### **Creating emission models of exhaust emissions**

Data for individual emission classes of the tested vehicles for model development have been aggregated into the main groups EURO2, EURO3, EURO4, EURO5, and EURO6.

Emission models for CO_2_, CO, THC, and NO*x* were created in the Regression Learner application, Matlab R2018b. The Regression Learner application gives the opportunity to choose a statistical method to find the most suitable regression model for the case being studied. Among the methods used, we can distinguish standard linear regression and more advanced ones, such as, regression trees and support vector technologies. For the analyzed exhaust emission data, the method of regression tree models was used, particularly boosted trees, due to the best results obtained. Boosted regression trees combine the strengths of two algorithms: regression trees (models that relate a response to their predictors by recursive binary splits) and boosting (an adaptive method for combining many simple models to give improved predictive performance). They have no need for prior data transformation or elimination of outliers, can fit complex nonlinear relationships, and automatically handle interaction effects between predictors (Elith et al. 2008). Unfortunately, it is infeasible to write down an equation of a tree-based regression model, since it is a combination of trees and each grown on the residuals of the previous tree; therefore, prediction is accomplished by weighting the ensemble outputs of all regression trees (Alzahabi et al. 2017, Ye et al. 2009).

In the development of models, the cross-validation method was also used. Cross-validation is a resampling procedure used to evaluate machine learning models on a limited data sample. The best general recommendation at present is to use five- or tenfold cross-validation (Hastie et al. 2009). With fivefold cross-validation, the data is randomly split into 5 equal portions (fivefold), use fourfold to train the model, and use remaining onefold to test. Results of cross-validation showed that for researched data, it is better to use 10-fold to obtain better accuracy of models.

The main novelty of the developed emission models is the methodology of obtaining them, which is based on data from the areas surrounding the roundabouts. It causes that their later use is more accurate for the needs of microstructural objects, in this case, roundabouts, so that obtained emission results may be more useful, e.g., for the needs of designing a new type of roundabouts as well as pedestrian crossing locations, in view of environmental impact.

**Fig. 6:**
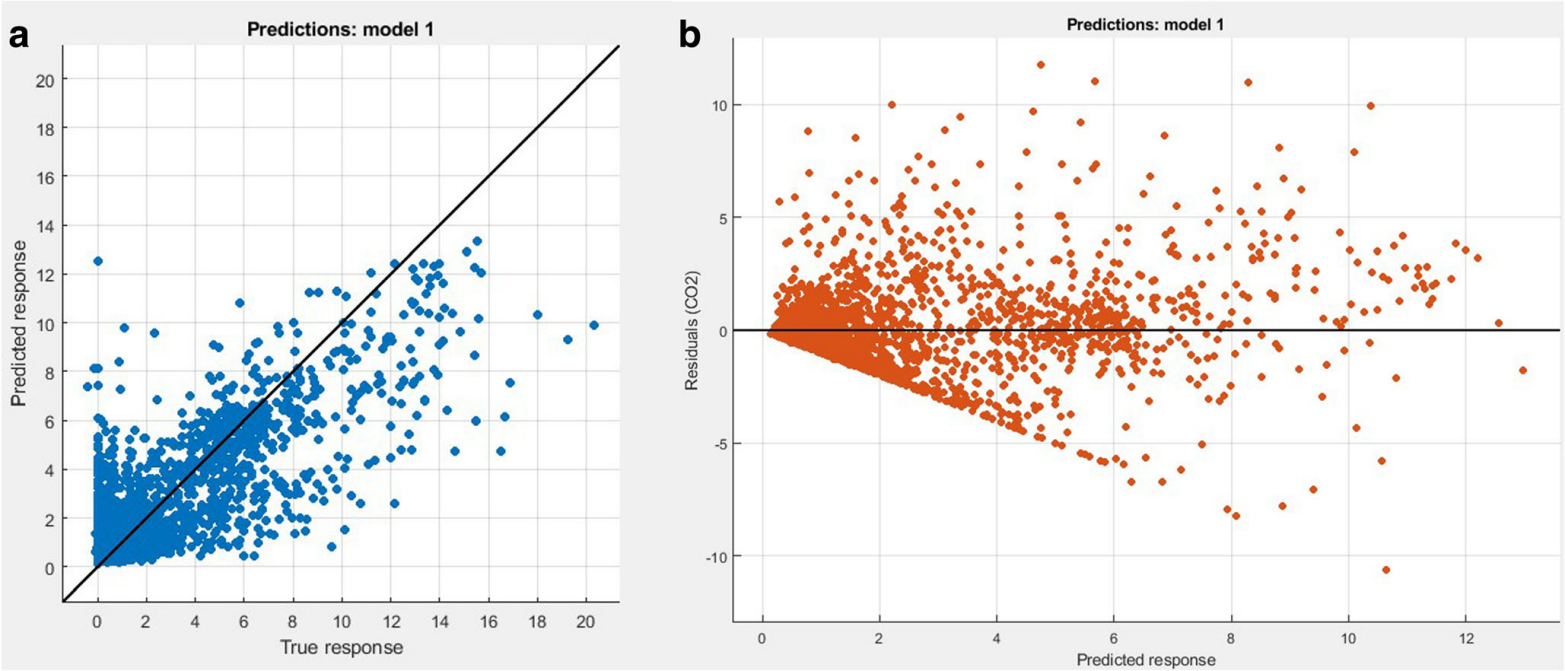
Example graphs of **a** predicted vs true response and **b** residuals vs. predicted response for CO_2_ of EURO4

Figure 6 presents plots of predicted vs true response plot and residuals for CO_2_ of EURO4. Generally based on plots and high value of the coefficient of determination, it suggests that models are detailed enough. The residua chart confirms that models can be respected as reliable because the data do not form any patterns and are distributed in such a way that their clusters go to the center of the graph. Values of *R*^2^ coefficient for all models are presented in Table 4.

**Table 4:** The coefficient of determination *R*^2^ for created emission models

Emission class/fuel	Exhaust component			
	CO_2_	CO	THC	NO*x*
EURO2/petrol	0.74	0.51	0.81	0.53
EURO3/petrol	0.68	0.49	0.75	0.61
EURO3/LPG	0.78	0.62	0.62	0.59
EURO4/petrol	0.81	0.59	0.82	0.73
EURO5/petrol	0.72	0.69	0.78	0.71
EURO6/petrol	0.80	0.45	0.63	0.54
EURO6/LPG	0.84	0.52	0.59	0.73
EURO6/diesel	0.82	0.62	0.65	0.68

The coefficient of determination is calculated based on the formula:

1 $$ {R}^2=\frac{S{S}_M}{S{S}_T}=\frac{\sum_{t=1}^n{\left({\widehat{y}}_t-\overline{y}\right)}^2}{\sum_{t=1}^n{\left({y}_t-\overline{y}\right)}^2} $$

where *R*^2^ is the coefficient of determination, SS_M_ is the sum of squares for the model, SS_T_ is the sum of squares total, *y*_t_ is the actual value of the dependent variable, ${\widehat{y}}_t$ is the predicted values of the dependent variable, and $\overline{y}$ is the average value of the actual dependent variable.

The coefficient of determination informs about what part of the variability of the explanatory variable was explained by the model. It accepts values between 0 and 1. Some sources say that the model fit is better, the *R*^2^ value is closer to one, but it is incorrect, because many observation data causes the *R*^2^ value to decrease, then the model assessment should be performed based on other model validation parameters.

### **Validation of the obtained models**

The assessment of the correctness of the model’s operation is the most important part of developing the models because it allows to the state to what extent the prepared model meets the assumed task. Validation of the obtained emission models was carried out based on the results of instantaneous emission, using data that were not used for earlier calibration of the model. Two widely used error estimation coefficients were used to validate the obtained models: RMSE (root mean square error) and MAE (mean absolute error). They are calculated based on equations:

2 $$ \mathrm{RMSE}=\sqrt{\frac{1}{n}}{\sum}_{t=1}^n{\left({y}_t-{y}_t^P\right)}^2 $$

3 $$ \mathrm{MAE}=\frac{1}{n}\;{\sum}_{t=1}^n\mid {y}_t-{y}_t^P\mid $$

where *n* is the number of samples, *y*_t_ is the forecast, and ${y}_t^P$ is the observed values.

The results of presented equations for obtained models are shown in Tables 5 and 6.

**Table 5:** RMSE mean square error element for created emission models

Emission class/fuel	Exhaust component			
	CO_2_ (g/km)	CO (g/km)	THC (10^−3^) (g/km)	NO*x* (10^−2^) (g/km)
EURO2/petrol	1.63	0.066	0.30	0.62
EURO3/petrol	1.52	0.012	0.50	0.65
EURO3/LPG	1.56	0.049	0.47	0.92
EURO4/petrol	1.72	0.167	0.49	1.27
EURO5/petrol	1.40	0.117	0.18	1.47
EURO6/petrol	1.15	0.105	0.27	1.50
EURO6/LPG	1.52	0.097	0.71	0.98
EURO6/diesel	1.12	0.103	0.82	1.12

**Table 6:** Absolute error MAE for created emission models

Emission class/fuel	Exhaust component			
	CO_2_ (g/km)	CO (g/km)	THC (10^−3^) (g/km)	NO*x* (10^−3^) (g/km)
EURO2/petrol	1.21	0.03	0.31	0.36
EURO3/petrol	1.41	0.03	0.28	0.29
EURO3/LPG	1.55	0.05	0.35	0.41
EURO4/petrol	1.63	0.04	0.26	0.28
EURO5/petrol	1.78	0.04	0.11	0.24
EURO6/petrol	1.12	0.06	0.16	0.35
EURO6/LPG	1.25	0.03	0.18	0.42
EURO6/diesel	1.18	0.02	0.21	0.18

**Fig. 7:**
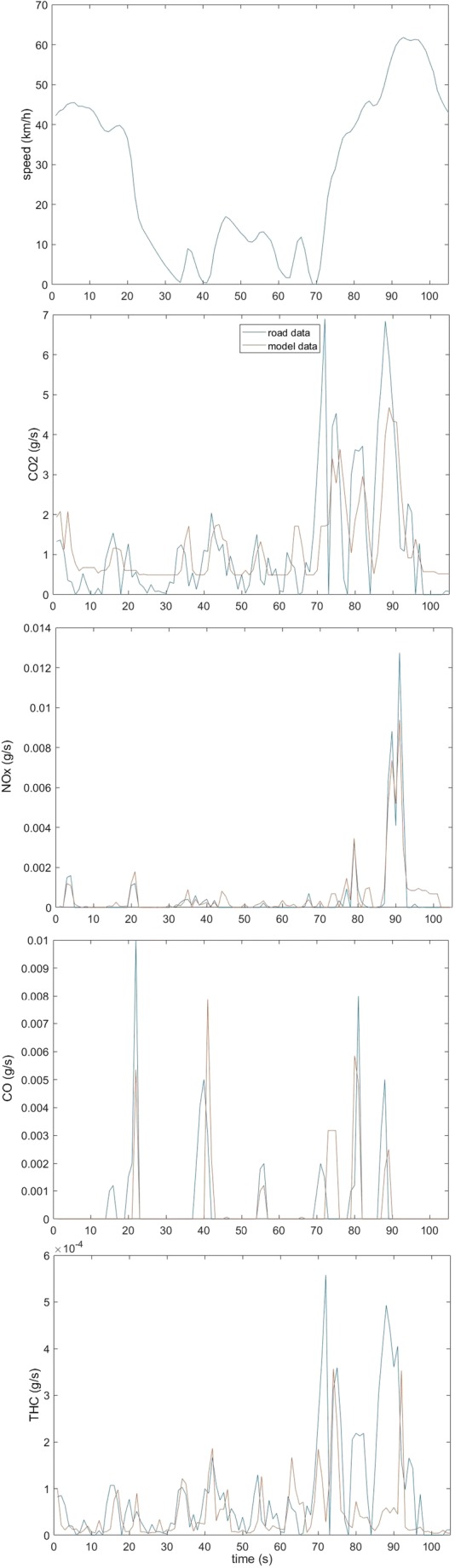
An example of instantaneous CO_2_, THC, CO, and NO*x* emissions in the comparison of measured values with the model-based results from the roundabout for EURO6 standard

Figure 7 presents examples of instantaneous CO_2_, THC, and CO emission charts for EURO6 class, passing twice through one of the researched roundabouts. Based on the results, it can be noticed that the obtained models to a good extent reflect the real emission of the tested exhaust components for roundabout objects. In the small emission phases of exhaust components, a decrease in the value of emissions from models can also be noticed. However, in the course of the growing real values of emission, emissions from models are also growing, but, for some temporary high values, the results obtained from the models are underestimated. Also, some inaccuracies can be noticed in the CO estimation because the graph shows instantaneous actual emission values when no CO emission was calculated from the model.

### **Application for calculating exhaust emissions based on obtained models**

**Fig. 8:**
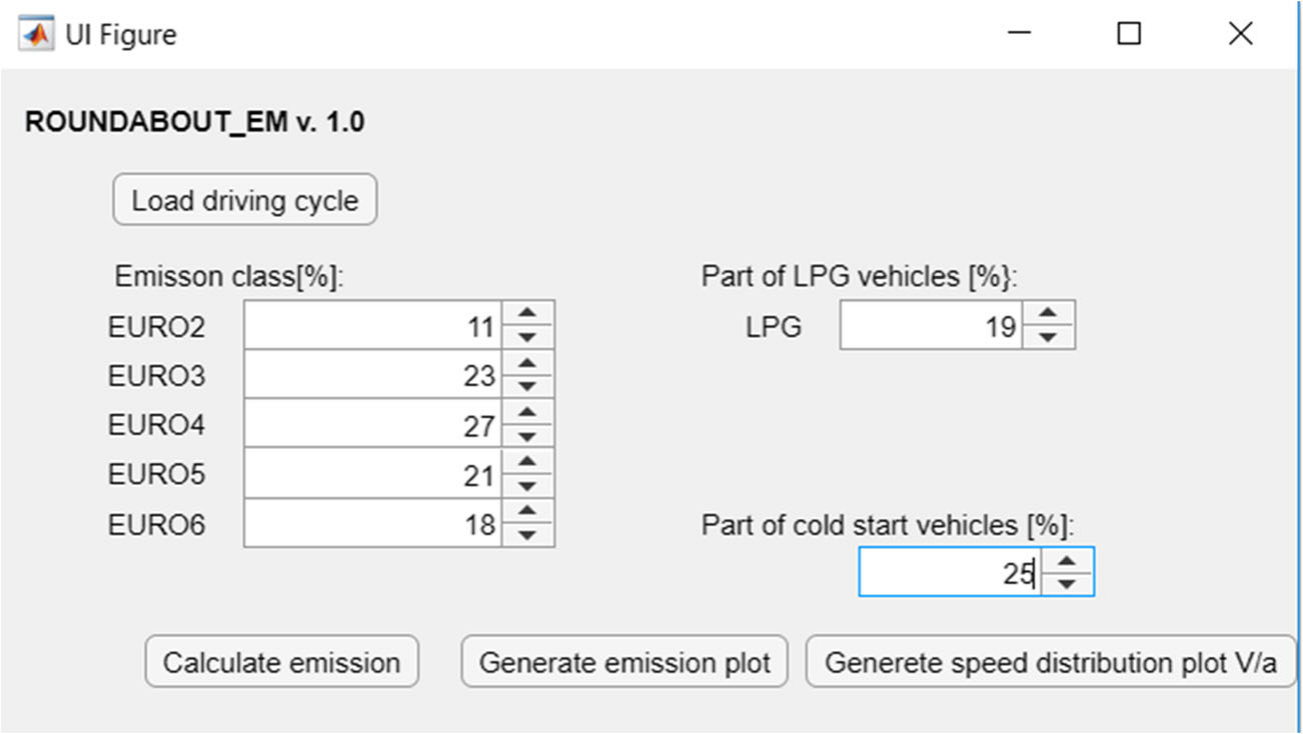
The main menu of the RoundaboutEM application

**Fig. 9:**
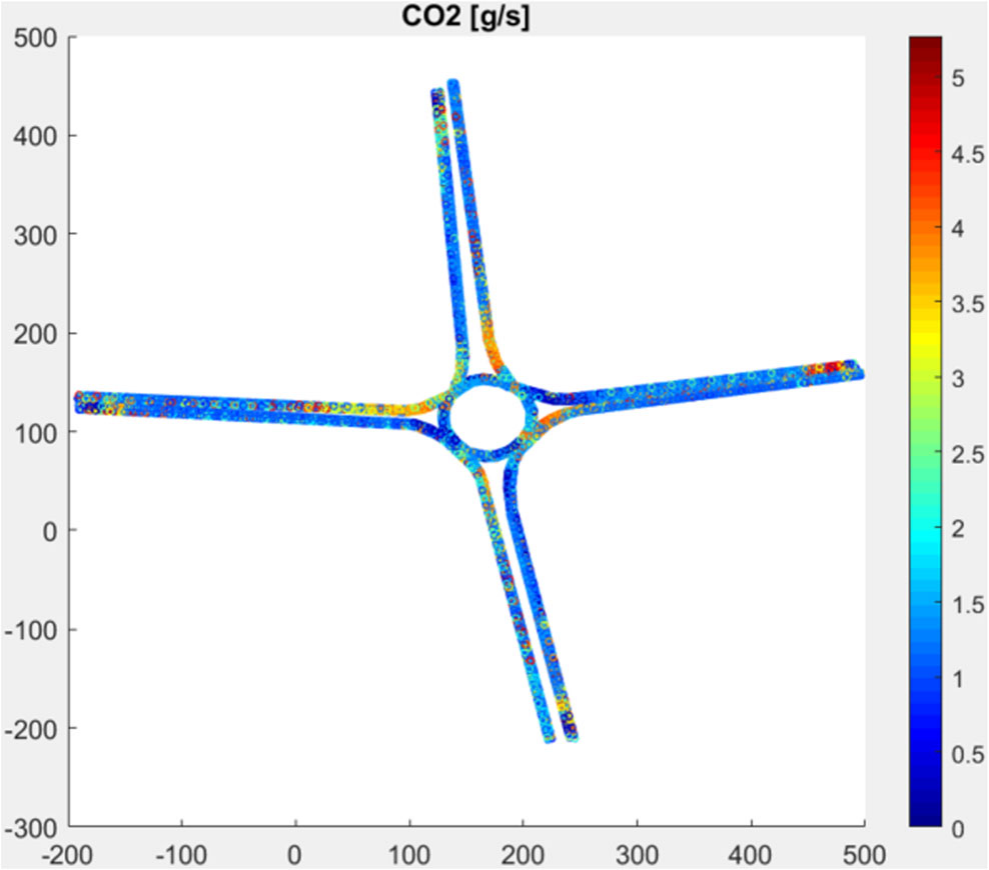
Example view of map emission for selected roundabout from RoundaboutEM application

Based on the developed emission models and App Designer application of the Matlab program, the RoundaboutEM application was prepared to calculate the emission of harmful exhaust pollution for roundabout objects. The application to work needs input data in the form of travel cycles and terrain gradient. To calculate the CO_2_, THC, CO, and NO*x* emissions, the Vissim program data can be used, but it is required to process them to the Matlab worksheet. The application also introduces the percentage share of emission classes of vehicles moving along the test road, the number of vehicles powered by LPG, and the percentage of the so-called cold start. A view of sample screenshots from the application is shown in Figs. 8 and 9.

Previous research has shown that models of exhaust emissions from vehicles should reflect the local vehicle structure, driving characteristics, and atmospheric conditions in order to give reliable results (Smit, 2013). The measurement methodology is also a key issue in conducting emission tests. So far, tests have been carried out using such methods as laboratory (station) tests, tunnel tests, remote sensing, and mobile measurement on the vehicle (Barth et al. 2000; Kuhns et al. 2004; Rogak et al., 1998; John et al., 1999; Yang and Yu, 2007; Elst et al. 2004). However, for the development of microscopic models, it is necessary to perform many road tests using PEMS systems. In recent years, the use of this system has significantly increased (Song et al., 2008; Li et al., 2004; Bielaczyc et al., 2018; He et al., 2019). Studies also show that the PEMS system is a reliable source for obtaining real-world emission data from vehicles compared to laboratory tests (Liu et al., 2010; Merkisz, Rymaniak, 2017). The results of exhaust emissions from road tests are significantly different from those that are performed during a standard test in stationary conditions (Joumard et al., 2000). In addition, vehicle emission tests have shown that current emission values have changed significantly over recent years with the introduction of new engines and exhaust after-treatment systems, which influenced the variability and increased the sensitivity of results (De Haan and Keller 2000). In order to assess the impact of changes in road traffic, e.g., drivers’ behavior on speed limits, traffic lights, and an occurrence of intersections, it is necessary to develop special models that will give a reliable emission prediction.

## **Conclusions**

The conducted research allows us to formulate the following conclusions:

- With a large number of vehicles wanting to drive roundabouts at the same time, they queue up, which causes a lot of stops and accelerations. The conditions of congestion at the inlets to the roundabouts result in prolonged time losses, long queues, and changes in vehicle speed cycles.
- The occurrence of these phenomena has a large impact on the quality of air in the roundabout area. Therefore, it is important to create calculation models of exhaust emissions from vehicles, also for detailed purposes, for example solutions for roundabouts, to find out where the roundabouts reach the largest accumulation of pollutants, which will allow for better placement of, e.g., pedestrian crossings, to reduce the impact of fumes on the health of passers-by.
- The use of created models could also contribute to the selection of the most environmentally friendly roundabout solution for traffic conditions prevailing in a given area.
